# *TAGLN* expression is upregulated in NF1-associated malignant peripheral nerve sheath tumors by hypomethylation in its promoter and subpromoter regions

**DOI:** 10.3892/or.2014.3379

**Published:** 2014-08-04

**Authors:** GUN-HOO PARK, SU-JIN LEE, HYUNE YIM, JAE-HO HAN, HYON J. KIM, YOUNG-BAE SOHN, JUNG MIN KO, SEON-YONG JEONG

**Affiliations:** 1 Department of Medical Genetics, Ajou University School of Medicine, Suwon, Republic of Korea; 2 Department of Biomedical Sciences, Ajou University School of Medicine, Suwon, Republic of Korea; 3 Department of Pathology, Ajou University School of Medicine, Suwon, Republic of Korea; 4 Department of Pediatrics, Seoul National University Children’s Hospital, Seoul, Republic of Korea

**Keywords:** neurofibromatosis type 1, malignant peripheral nerve sheath tumor, transgelin, *TAGLN* gene, epigenetics, DNA methylation

## Abstract

Neurofibromatosis type 1 (NF1) caused by *NF1* gene mutation is a commonly inherited autosomal dominant disorder. Malignant peripheral nerve sheath tumors (MPNSTs), a type of aggressive sarcoma, are a major cause of mortality in NF1 patients. The malignant transformation of benign plexiform neurofibromas (PNs) to MPNSTs is a marked peculiarity in NF1 patients, yet the pathogenesis remains poorly understood. We found that an actin-associated protein transgelin (SM22) was highly expressed in *NF1*-deficient MPNST tissues compared to *NF1*-deficient PN tissues using immunohistological staining and primary cultured MPNST cells in western blot analysis. We further found that this transgelin upregulation was caused by increased transcriptional expression of the *TAGLN* gene encoding transgelin. Comparison of DNA methylation values in the promoter and subpromoter regions of the *TAGLN* gene in three types of *NF1*-deficient primary-cultured cells, derived from an NF1 patient’s normal phenotype, a benign PN and MPNST tissues, revealed that the *TAGLN* gene was hypomethylated in the MPNST cells. Next, to determine the functional role of transgelin in MPNST pathogenesis, we manipulated the *TAGLN* gene expression and investigated the alteration of the RAS-mitogen-activated protein kinase (MAPK) signaling pathway in the normal-phenotypic and malignant tumor cells. The downregulation of *TAGLN* expression in *NF1*-deficient MPNST tumor cells through the treatment of the small interfering RNA resulted in a decrease in the RAS activation (GTP-RAS) and the downstream ERK1/2 activation (phosphorylated ERK1/2), while the overexpression of *TAGLN* in normal-phenotypic *NF1*-deficient cells caused an increase in RAS and ERK1/2 activation. These results indicate that upregulation of transgelin caused by hypomethylation of the *TAGLN* gene is closely involved in tumor progression in NF1.

## Introduction

Neurofibromatosis type 1 (NF1; OMIM #162200) is a commonly inherited autosomal dominant disorder, occurring with an incidence of 1 in 3,000–3,500 individuals worldwide (1,2). NF1 is caused by loss of function mutations in the *NF1* gene (GenBank gene ID: NG_009018.1), which encodes a GTPase-activating protein, neurofibromin (3). The major clinical features of NF1 include cafe-au-lait spots, freckling of the axillary or inguinal region, Lisch nodules, optic nerve glioma, bone dysplasias and nerve sheath tumors in the peripheral nervous system, including benign cutaneous neurofibromas, benign plexiform neurofibromas (PNs), and malignant peripheral nerve sheath tumors (MPNSTs) (4).

Since neurofibromin is a major RAS inactivator and plays a role as a tumor suppressor, the lack of neurofibromin resulting from *NF1* mutation causes disruptions in the RAS-mitogen-activated protein kinase (MAPK) and PI3K-AKT-mTOR signaling pathways, which is implicated in the tumorigenesis and tumor progression of PN to MPNST (5). MPNST, also called malignant schwannoma or neurofibrosarcoma, develops in 8–13% of NF1 patients (6), and it represents a major cause of mortality in NF1 patients (7). Thus, the pathogenesis of the malignant transformation of PN to MPNST in NF1 patients has attracted considerable attention (8,9). However, the exact molecular mechanisms of MPNST pathogenesis remain unclear.

Bi-allelic loss of *NF1* gene function (*NF1*^−/−^) due to the somatic loss of heterozygosity (LOH) of the *NF1* gene has been reported to be essential for MPNST development (10). However, LOH at the *NF1* locus was also found in PNs (11), suggesting that other genetic and/or epigenetic alterations may be involved in tumor progression in NF1. Emerging evidence has suggested that additional driver gene mutations and/or expression alterations contribute to the tumor progression of PNs to MPNSTs (5). Mutations in tumor suppressor genes, including *TP53*, *CDKN2A* and *RB* are commonly identified in MPNSTs (12–14). It was also reported that several cell-cycle and signaling regulation genes, including *CDKN2A*, *CD44*, *CXCR4*, *EGFR*, *HGF*, *MET*, *PDGFR*, *PDGFRA*, *RB1*, *SOX9* and *TP53*, are deregulated in MPNSTs (15,16). In addition, many differentially expressed genes between benign neurofibromas and MPNSTs, such as *KIT*, *ERBB2*, *MET*, *TGFB1*, *HGF*, *TNXB*, *TNC*, *MTOR*, *TSC2*, *PTEN*, *BCL2L1*, *CDK4*, *FOXM1*, *BUB1B*, *PBK* and *NEK2*, have been identified in comparison studies with immunohistochemical, immunoblotting, microarray-based, quantitative reverse transcription PCR-based or comparative genomic hybridization array-based analyses (17–24).

DNA methylation alterations in cancer-related genes and microRNA (miRNA) dysregulation have been critically implicated in the development and progression of many cancers (25,26). Genes encoding miRNA can be epigenetically regulated by DNA methylation or specific histone modifications (26). Recently, the emerging role of miRNAs in the pathogenesis of NF1 tumorigenesis and MPNST development were reported (27). Genome-wide miRNA profiling analyses found a large number of dysregulated miRNAs in MPNSTs, such as miR-34a against *TP53* (28), miR-301a, miR-19a and miR-106b against *PTEN* (29) and miR-21 against *PDCD4* (30). However, only a few DNA methylation studies on NF1 have been reported. In studies of miRNAs, genome-wide DNA methylation analysis may be a new strategy for understanding the etiology of MPNST development in NF1.

In the present study, we reported a *TAGLN* gene encoding an actin-binding transgelin protein as a novel candidate that plays a critical role in NF1-associated MPNST pathogenesis. *TAGLN* was upregulated in MPNST tissues and cells derived from NF1 patients. Notably, we found that this upregulation was caused by an alteration of the DNA methylation in the *TAGLN* genes in MPNSTs. Manipulation of *TAGLN* expression in the *NF1*-deficient cells demonstrated the key role of transgelin in MPNST pathogenesis. This finding may provide insight into the underlying mechanisms of somatic tumor progression in NF1 on the epigenetic level.

## Materials and methods

### Tumor tissue samples

We used the tumor tissues of eight patients diagnosed with NF1 at the Ajou University Hospital according to NF1 diagnostic criteria (31) (Table I). The PN and MPNST tumor samples were obtained through surgical resection at the Ajou University Hospital. In the case of patients P7 and P8, two tumor specimens obtained from surgeries at two different times were used in the present study. The present study was approved by the Institutional Review Board Committee of the Ajou University School of Medicine.

### Primary tissue cultured cells and cell lines

We used three types of previously established primary tissue-cultured *NF1*-deficient cells: normal phenotype tissues (PC-N), benign PN tissues (PC-B) and the MPNST tissues (PC-M) of NF1 patient P8 (32). The cellular characteristics, including GTP-RAS activity and its downstream effectors in the three cell types, were previously demonstrated (32). Cells were grown in Dulbecco’s modified Eagle’s medium (DMEM) supplemented with 15% fetal bovine serum (FBS) (both from HyClone Laboratories, Logan, UT, USA), penicillin (100 U/ml) and streptomycin (100 μg/ml). Cells were used from passages 5 through 10. The *NF1*-deficient MPNST cell lines, sNF02.2 and sNF96.2, were purchased from the American Type Culture Collection (Manassas, VA, USA) and grown in DMEM media supplemented with 10% FBS. All cultured cells were incubated at 37°C in a humidified atmosphere containing 5% CO_2_.

### Hematoxylin and eosin (H&E) staining and immunohistochemistry (IHC)

The tumor tissue specimens from eight patients with NF1 were formalin-fixed and embedded in paraffin wax for pathological evaluation by H&E staining and IHC. Serial 3 μm sections were prepared on glass using a cryostat and the slides were stained with H&E. For IHC, the blocks were cut at 10-μm thickness and adhered to glass slides. They were deparaffinized in xylene and rehydrated with graded ethanol, which was followed by antigen retrieval in boiling 10 mM citrate buffer (pH 6.0) for 4 min. Immunostaining was carried out using the UltraVision LP Detection System and the HRP Polymer and DAB Plus Chromogen (Thermo Fisher Scientific, Fremont, CA, USA) according to the manufacturer’s instructions. Briefly, the sections were incubated with Ultra V Block for 5 min at room temperature to reduce the nonspecific background, and they were then treated with hydrogen peroxide to block endogenous peroxidase activity. The sections were incubated with the primary antibody for 1 h at room temperature and then incubated with HRP polymer for 20 min at room temperature. The reaction product was visualized with the DAB chromogen. Pathological evaluation was performed under light microscopy. Anti-transgelin and anti-S100 antibodies were purchased from Thermo Science (Rockford, IL, USA) and Abcam (Cambridge, UK), respectively.

### Plasmid constructs and small interfering RNAs (siRNAs)

Full-length human *TAGLN* cDNA was amplified by reverse transcription polymerase chain reaction (RT-PCR) using the primers: 5′-AGTGCAGTCCAAAATCGAGAAG-3′ and 5′-CTTGCTCAGAATCACGCCAT-3′, which were from the total RNAs of the human skin tissue-cultured fibroblast cells. The cDNAs were subcloned into the pcDNA3.1(−) vector (Clontech, Palo Alto, CA, USA) using the *Xho*I and *Bam*HI restriction enzyme sites. The siRNAs were synthesized by Genolution Pharmaceuticals, Inc. (Seoul, South Korea). The target sequences for the siRNAs were: 5′-CCAAAATCGA GAAGAAGTATT-3′ for the *TAGLN* gene, and 5′-CCTACGC CACCAATTTCGT-3′ for the non-specific scramble siRNA control. Cell transfection of the siRNAs and plasmid constructs was conducted using Lipofectamine RNAiMAX (Invitrogen, Carlsbad, CA, USA) and Lipofectamine 2000 (Invitrogen), respectively, according to the manufacturer’s instructions.

### Reverse transcription-PCR (RT-PCR)

Total RNAs were isolated from the cultured cells using TRIzol reagent and they were treated with RNase-free DNase I (both from Invitrogen) to avoid amplification of the genomic DNA, and were subsequently reverse transcribed using the RevertAid™ H Minus First Strand cDNA Synthesis kit (Fermentas, Burlington, ON, Canada) with the oligo(dT)_15–18_ primer. PCR amplification was carried out using the Ex-Taq DNA polymerase (Takara, Shiga, Japan) at an annealing temperature of 60°C for 25 cycles. The gene specific primers used were: 5′-AGTGCAGTCCAA AATCGAGAAG-3′ and 5′-CTTGCTCAGAATCACGCCAT-3′ for the *TAGLN* gene; and 5′-TGTTGCCATCAATGACCC CTT-3′, and 5′-CTCCACGACGTACTCAGCG-3′ for the *GAPDH* gene (a control).

### Western blot analysis

Cultured cells were lysed in RIPA buffer [150 mM NaCl, 1% Nonidet P-40, 0.5% sodium deoxycholate, 0.1% sodium dodecyl sulfate (SDS) and 50 mM Tris buffer, pH 8.0]. The proteins were heated at 100°C for 10 min and analyzed by SDS-polyacrylamide gel electrophoresis on 8–12% polyacrylamide gels. The proteins were electroblotted onto PVDF membranes (Millipore, Milford, MA, USA). The membrane blots were blocked with 5% (w/v) non-fat dried milk, incubated with primary and secondary antibodies, and then visualized with the enhanced chemiluminescence western blotting detection system (WEST-ZOL plus; iNtRON Biotechnology, Daejeon, Korea). Anti-transgelin was purchased from Abcam, while the anti-extracellular regulated kinase (ERK)1/2 and anti-phosphorylated ERK1/2 antibodies were purchased from Cell Signaling Technology (Danvers, MA, USA). Anti-α-tubulin, HRP-conjugated goat anti-rabbit IgG and HRP-conjugated goat anti-mouse IgG antibodies were purchased from Santa Cruz Biotechnology (Santa Cruz, CA, USA).

### Ras activation assay

Ras activation was assayed using the RAS activation assay kit (Upstate Biotechnology, Lake Placid, NY, USA). Briefly, cells were lysed in lysis buffer (25 mM HEPES pH 7.5, 150 mM NaCl, 1% Igepal CA-630, 10 mM MgCl_2_, 1 mM EDTA, 2% glycerol, 25 mM NaF and 1 mM Na_3_VO_4_) on ice and centrifuged at 14,000 × g for 5 min at 4°C. A total of 300 μg cellular lysates were incubated with Raf-RBD agarose beads at 4°C for 1 h. The beads were washed 3 times with 1 ml of ice-cold lysis buffer, resuspended in 2X Laemmli sample buffer, boiled and separated on SDS-PAGE gels, which was followed by western blot analysis using an anti-ras antibody.

### DNA methylation analysis

DNA methylation analysis was performed using the Infinium HumanMethylation27 BeadChip (Illumina Inc., San Diego, CA, USA; catalog no: WG-311–1201), which contains 27,578 CpG loci located in the promoter and subpromoter regions (5′-UTR, exon 1 and intron 1) of 14,495 human RefSeq genes, as previously described (33). Genomic DNA was isolated from cells using the QIAamp DNA Mini kit (Qiagen, UK). The bisulfite conversion of genomic DNA (1 μg) was carried out using the EZ DNA Methylation-Gold kit (Zymo Research, Orange, CA, USA). Bisulfite-converted DNA was amplified and hybridized to the HumanMethylation27 BeadChip according to the manufacturer’s standard protocols. Each interrogated locus is represented by specific oligomers linked to two bead types: one representing the sequence for methylated DNA (M) and the other for unmethylated DNA (U). For each specific CpG island region, the methylation status is calculated from the intensity of the M and U alleles, as the ratio of the fluorescent signals β = Max(M,0)/[Max(M,0)+Max(U,0)+100]. The DNA methylation β-value is a way to represent the scores of DNA methylation. Quantitative scores of DNA methylation levels range from 0 (methylation absent) to 1 (completely methylated).

## Results

### Upregulation of transgelin in MPNST tissues and primary MPNST cells from NF1 patients

Based on the analysis of the RT-PCR-based differential display data of the normal phenotype, benign PN and MPNST tissues of an NF1 patient, we previously found transgelin (SM22) to be significantly upregulated in MPNST tissues (34). Here, we sought to confirm this finding in more tumor tissues of NF1 patients using IHC analysis. The clinical features and genotypes of the eight NF1 patients analyzed in the present study are summarized in Table I. First, the PN and MPNST tumor specimens of eight NF1 patients were evaluated using histopathological analysis with H&E staining (Table I). Since Schwann cells are the primary pathogenic cell source in PN and MPNST tumors, we further evaluated tumor specimens through IHC analysis using the Schwann cell lineage marker S100 (Table I).

**Figure 1:**
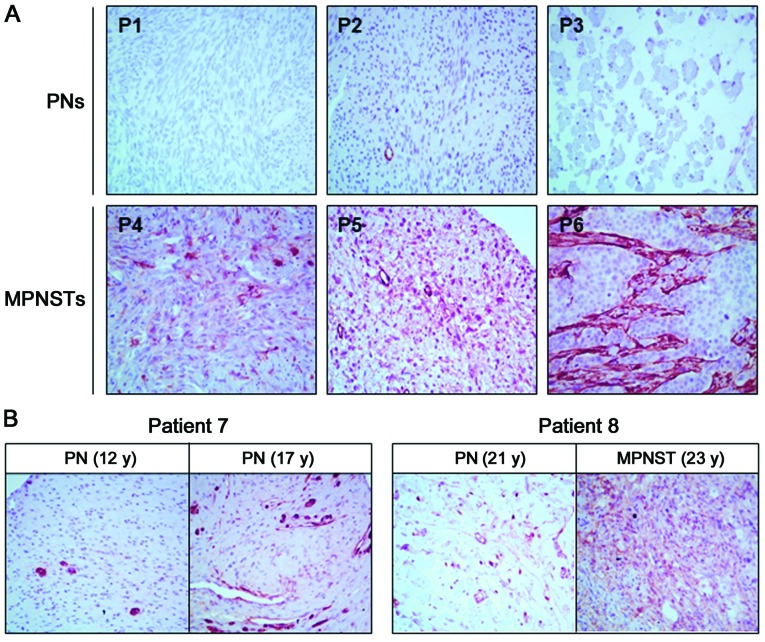
Immunohistochemical staining of transgelin in the plexiform neurofibroma (PN) and malignant peripheral nerve sheath tumor (MPNST) tissues from patients with NF1. (A) Comparison of transgelin levels between tumor tissue sections of PNs (patients P1–P3) and MPNSTs (patients P4–P6). (B) Comparison of transgelin levels in tumor tissue sections from the same patient that were taken at different times. NF1, neurofibromatosis type 1.

We compared the transgelin protein levels between the PN and MPNST tumor tissues using IHC analysis. The expression levels of transgelin were higher in the MPNST tissues (P4–P6) than in PN tissues (P1–P3) (Fig. 1A). Next, to investigate whether the transgelin expression level was altered in accordance with tumor progression, we compared the transgelin levels in the two tumor tissues resected at different times from the same patient. In patient P7, different transgelin levels were observed in two PN tumors with a five-year interval; the latter showed higher transgelin levels than the former (Fig. 1B). In patient P8, we compared the PN tumor from the first surgery and the MPNST tumor from the second surgery (a 2-year interval) and found significantly higher transgelin expression in the MPNST tumor than in the PN tumor (Fig. 1B).

**Figure 2:**
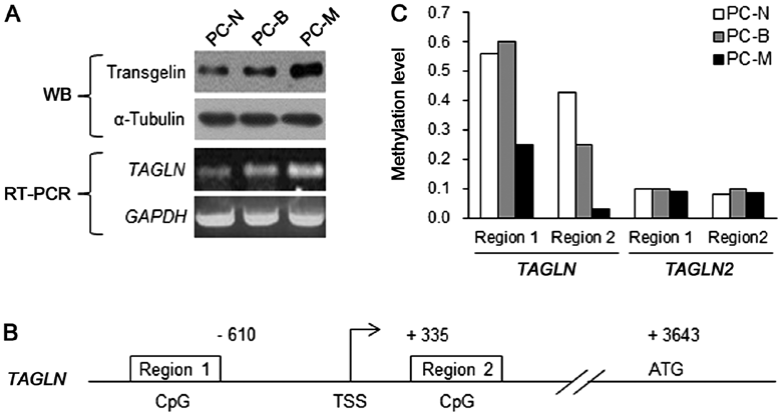
Comparison analyses of the gene expression level and the DNA methylation level of *TAGLN* in three types of primary *NF1*-deficient cells. Primary-cultured normal-phenotypic cells (PC-N), benign PN cells (PC-B) and MPNST cells (PC-M) from NF1 patient P8 were used in this study. (A) Western blot analysis of transgelin and RT-PCR analysis of *TAGLN* gene in the three primary cells. (B) Structure of the promoter and subpromoter regions of the *TAGLN* gene. Two CpG island regions (region 1 and 2) based on the HumanMethylation27 BeadChip data, transcription start site (TSS), and translation initiating ATG site are indicated with the corresponding cDNA position. (C) DNA methylation status of the *TAGLN* and *TAGLN2* genes in the three primary cells. DNA methylation β-values of the *TAGLN* and *TAGLN2* genes from the HumanMethylation27 BeadChip microarray are represented as scores from 0 (no methylation) to 1 (complete methylation). NF1, neurofibromatosis type 1; PN, plexiform neurofibroma; MPNST, malignant peripheral nerve sheath tumor.

Subsequently, we confirmed this result in the primary *NF1*-deficient cells from NF1 patient P8 (32): normal-phenotypic cells (PC-N), benign PN cells (PC-B) and MPNST cells (PC-M). Western blot analysis revealed that transgelin expression gradually increased from PC-N to PC-M (Fig. 2A). To determine whether the differential expression of transgelin among the cells was responsible for the changes in the transcriptional expression of the *TAGLN* gene, we examined the *TAGLN* mRNA levels by RT-PCR. Consistent with the results for the protein level, the mRNA expression level of *TAGLN* also gradually increased from PC-N to PC-M (Fig. 2A).

### Hypomethylation in the promoter and subpromoter regions of the TAGLN gene in primary MPNST cells from NF1 patients

Since DNA methylation alteration of the *TAGLN* gene was reported in cancers (35,36), we attempted to investigate the *TAGLN* gene methylation level in the primary *NF1*-deficient cells. Using the HumanMethylation27 BeadChip microarray, we performed genome-wide DNA methylation analysis. Following analysis of the methylation data of 27,578 CpG loci located in the promoter and subpromoter regions of 14,495 human genes (data not shown), we intensively examined the methylation status of two CpG island regions in the *TAGLN* gene (Fig. 2B). We also examined the DNA methylation status of the *TAGLN*2 gene, which is closely related to *TAGLN* (37), as a comparison control. The results revealed that both CpG island regions, regions 1 and 2, in the *TAGLN* gene were less methylated in the primary PC-M cells than in the PC-N and PC-B cells (Fig. 2C). The DNA methylation level in region 2 of the *TAGLN* gene gradually decreased from PC-N to PC-M. In particular, region 2 of the PC-M cells was extremely hypomethylated. In contrast, the DNA methylation status of the *TAGLN*2 gene was not different in the three types of cells (Fig. 2C). These results indicate that the upregulation of transgelin in the MPNSTs was caused by the hypomethylation of the *TAGLN* gene.

### Involvement of transgelin in RAS and ERK1/2 activation in primary MPNST cells and MPNST cell lines

**Figure 3:**
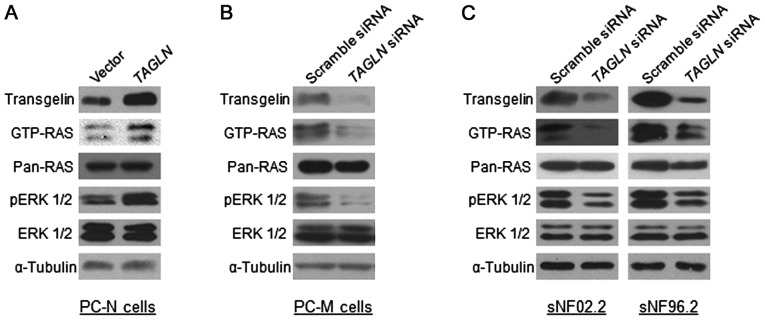
Transgelin level-dependent alterations of RAS and ERK1/2 activation in *NF1*-deficient primary cells and MPNST cell lines. (A) Overexpression of *TAGLN* in primary-cultured normal-phenotypic cells (PC-N) from NF1 patient P8. PC-N cells were transfected with a pCDNA3.1 plasmid vector or a *TAGLN* construct and then incubated for 24 h. (B) Knockdown of *TAGLN* using small interfering RNAs (siRNAs) in primary-cultured MPNST cells (PC-M) from NF1 patient P8. PC-M cells were treated with *TAGLN* siRNAs (50 nM) or non-specific scramble control siRNAs (50 nM) and incubated for 72 h. (C) Knockdown of *TAGLN* using siRNAs in MPNST cell lines, sNF02.2 and sNF96.2. Cells were treated with *TAGLN* siRNAs (50 nM) or nonspecific scramble control siRNAs (50 nM) and incubated for 72 h. The protein levels of the transgelin, GTP-RAS, total RAS (pan-RAS), phosphorylated ERK1/2 (pERK1/2), ERK1/2 and α-tubulin were determined by western blotting. The α-tubulin protein level was used as an internal control. NF1, neurofibromatosis type 1; MPNST, malignant peripheral nerve sheath tumor.

To examine the functional role of transgelin in MPNST pathogenesis, we manipulated the *TAGLN* gene expression and investigated if the RAS signaling was altered according to the transgelin expression level in the normal-phenotypic cells and malignant tumor cells. Overexpression of *TAGLN* in the primary PC-N cells caused an increase in Ras activation (GTP-RAS) and the downstream ERK1/2 activation (phosphorylated ERK1/2, pERK1/2) (Fig. 3A). In contrast, the downregulation of *TAGLN* expression in the primary PC-M cells through the treatment of the short interfering siRNAs (siRNAs) resulted in a decrease in RAS and ERK1/2 activation (Fig. 3B). Next, we carried out a further downregulation experiment in two Schwann-like MPNST cell lines, sNF02.2 (*NF1*^+/−^) and sNF96.2 (*NF1*^−/−^) (38), that express a high level of transgelin (Fig. 3C). Both cell lines treated with *TAGLN* siRNAs showed decreased GTP-RAS and pERK1/2, as in the primary PC-M cells (Fig. 3C). These results indicate that the transgelin level is closely involved with the Ras/Raf/Mek/Erk signaling pathway.

## Discussion

Aberrations of DNA methylation in cancer-related genes play a key role in cancer development (39). Hypermethylation of the promoter or first exon of tumor suppressor genes causes transcriptional silencing. In contrast, hypomethylation in proto-oncogenes induces transcriptional activation. Hypermethylation could explain the somatic loss of the tumor suppressor gene function without gene mutations in cancer. However, how hypomethylation contributes to carcinogenesis is less clear (40). In addition, DNA methylation is crucially involved in the dysregulation of miRNAs, which are small non-coding RNAs that act as post-transcriptional regulators of target gene expression, in cancer (41).

Only a few methylation studies have been conducted on NF1. A methylation study on the monozygotic twin pairs with NF1 that presented with several discordant features indicated that differences in the methylation patterns of the normal *NF1* allele in twins may result in *NF1* expression difference, thereby causing a modification of the NF1 phenotype (42). Comparing NF1 patients with a low number of cutaneous neurofibromas to those with a high number of cutaneous neurofibromas, using methylation-specific PCR and pyrosequencing, indicated that the promoter methylation of the mismatch repair *MSH2* gene in the blood cells of patients was significantly different (43). Another methylation study on normal Schwann cells and NF1-associated PN tumor samples reported that a low level of methylation in *NF1* gene promoters was found in PN tumors (44). In a methylation analysis of MPNSTs, although no significant methylation changes in the *NF1* gene promoter were found in the MPNST tissues (45), frequent *PTEN* promoter methylation was detected in *NF1*-associated MPNST tissues and *NF1*-deficient MPNST cell lines, although not in benign tumors (46).

In the present study, we found hypomethylation of the *TAGLN* gene in the primary MPNST cells and a high expression of transgelin in the MPNST tissues, primary MPNST cells and MPNST cell lines (Figs. 1 and 2). Since the methylation levels in CpG island region 2 of the *TAGLN* gene were in inverse proportion to the mRNA expression levels of the *TAGLN* gene in three types of primary cells (Fig. 2), we concluded that upregulated transgelin in the MPNSTs was a result of the hypomethylation of the *TAGLN* gene. Although the human promoter region of the *TAGLN* gene contains two CArG boxes, a binding site for the serum response factor and other transcription factor binding sites for AP-1 and SP1 (47), the epigenetic modification of *TAGLN* through DNA methylation was also reported to be important in the regulation of transgelin transcription (48). Contrary to our results, however, hypermethylation of *TAGLN* was found in hepatocellular and colorectal carcinoma (35,36).

Transgelin (SM22) is a 22-kDa actin-binding protein of the calponin family that is found abundantly in the smooth muscle tissues of adult vertebrates (49). Although the precise function of the protein remains unclear, transgelin is suggested to be involved in cell differentiation, cell migration, podosome formation, tissue invasion and matrix remodeling (50,51). Most previous studies reported that transgelin acted as a tumor suppressor (49). However, recent data have indicated that it has a pro-tumorigenic role (50). The upregulation of transgelin was observed in gastric and pancreatic cancers (52,53). Based on these controversial results, the pathological role of transgelin appears to be different between cancer types and could change during tumor progression (50).

Neurofibromas and MPNSTs consist mostly of Schwann cells and fibroblasts and they also contain other cell types, including perineural and mast cells, pericytes, endothelial and smooth muscle cells (54). Fibroblasts are known to express transgelin (53) and Schwann cells also express transgelin based on our data (Fig. 3C), indicating that two of the major cells forming MPNST tumors, Schwann cells and fibroblasts, express transgelin. Thus, the hyperexpression of transgelin in the MPNSTS in the present study may reflect the hyperexpression in these two types of cells, as well as in smooth muscle cells. Schwann cells are well known to contribute fundamentally to MPNST development (55). Fibroblasts are associated with cancer cells in all stages of cancer progression through the production of growth factors, chemokines and the extracellular matrix, and therefore fibroblasts are a key determinant in the malignant progression of cancer (56). It was reported that the upregulation of transgelin in stromal fibroblasts promoted gastric cancer cell migration and invasion by inducing the expression of matrix metalloproteinase-2 (MMP-2) (53). Further studies are required to elucidate how transgelin upregulation in these cells is implicated in tumor progression in NF1.

The molecular mechanisms of transgelin in cancer development remain poorly understood (49,50). The involvement of transgelin in transforming growth factor β (TGF-β) and MMP-9 has been reported (49). In addition, RAS-dependent and RAS-independent downregulation of transgelin in human breast and colon carcinomas has been reported (57). Although this study showed results opposite to our results, in that transgelin is downregulated in tumors, it is consistent with our results on how activation of the RAS-RAF-MEK-ERK signaling pathway antagonizes transgelin expression. Our data demonstrated that the upregulation of transgelin in normal-phenotypic primary cells by *TAGLN* overexpression caused an increase in RAS and ERK1/2 activation, while the downregulation of transgelin in MPNST primary cells and cell lines by siRNA treatment resulted in decreased RAS and ERK1/2 activation (Fig. 3). These results indicate that transgelin is closely involved in the RAS-MAPK signaling pathway.

Our data indicated that transgelin acts as a proto-oncogene rather than as a tumor suppressor gene in NF1-associated MPNST development, which suggests that transgelin may be a suitable target molecule to be inhibited for therapeutic treatment and/or prevention of MPNSTs. Pancreatic cancer patients with high transgelin expression showed a shorter 5-year overall survival rate than those with low transgelin expression (52). Since reduced transgelin expression was accompanied by significantly decreased RAS-MAPK signaling in MPNST cells, the inhibition of transgelin expression may attenuate MPNST development. The upregulation of the miR-145 miRNA enhanced transgelin expression (58); therefore, inhibitors of miR-145 could also be therapeutic drugs in MPNSTs. Gene-specific and genome-wide DNA methylation profiling may be useful biomarkers for diagnosis, risk assessment, prognosis, therapy and therapy-response prediction in cancer (59,60). Recent advances in the field of DNA methylation have begun to elucidate the underlying mechanisms of DNA methylation modification in cancers and to identify novel biomarker targets (60). The methylation profile of the *TAGLN* gene may be another biomarker that is useful for decision making with regard to the tumor progression of NF1-associated tumors.

## Figures and Tables

**Table I:** Clinical and histological characteristics of the patients with NF1.

Patient			Histological findings			Clinical features							
			
ID	Gender	Age at diagnosis	H&E	S100	Transgelin	Cafe-au-lait spots	Neurofibromas	Freckling	Optic glioma	Lisch nodule	Skeletal dysplasia	Family history	Genotype *NF1* gene mutation
P1	Male	59	Benign	+	+	Y	Y	Y	N	N	N	Y	N/A
P2	Male	42	Benign	+	+	Y	Y	Y	N	N	N	Y	N/A
P3	Female	5	Benign	+	+	Y	Y	Y	N	N	N	Y	N/A
P4	Male	39	Malignant	+	++	Y	Y	N	N	N	N	N	N/A
P5	Male	32	Malignant	+	++	Y	Y	Y	N	N	N	N	c.4861_4862 GT>AG
P6	Female	41	Malignant	+	++	Y	Y	N	N	N	N	N	N/A
P7	Male	12	Benign	+	+	Y	Y	Y	N	Y	Y	N	c.4537C>T
		17	Benign	+	++	Y	Y	Y	N	Y	Y	N	c.4537C>T
P8	Male	21	Benign	+	+	Y	Y	Y	N	Y	N	N	c.6792C>A
		23	Malignant	+	++	Y	Y	Y	N	Y	N	N	c.6792C>A

NF1, neurofibromatosis type 1; H&E, hematoxylin and eosin.
